# The Covid-19 Emergency and the Risk of Social Fragmentation in the Palermo Case

**DOI:** 10.3389/fsoc.2020.624160

**Published:** 2021-01-06

**Authors:** Antonia Roberta Siino

**Affiliations:** University of Bologna, Bologna, Italy

**Keywords:** Covid-19 emergency, Palermo, social fragmentation, technological gap, local communities, new challenges, inequalities, quality of life

## Abstract

The global Covid-19 crisis has shown its impact on all the aspect of life: health, social, economic, and politic. The attention mainly reserved to the economic and politic aspect musts also take into account the risk of social fragmentation. In which way this emergency has impact on communities where social fragilities were already existing? In which way, social dynamics are changed by the fear of the pandemic and the physical isolation imposed by governments to contain it. In which way local associations and citizen are coping the new challenges imposed by this emergency? In this article, the Sicilian context will be explored with a specific view on the Palermo capital. This Italian region has a peculiar and complex social asset determined by interrelated socio-economic phenomena such as mafia, unemployment and poverty. Fortunately, the existence of problematic and historical social phenomena has to face with the constant activities organized by local associations, civil society and authorities to fight them. There is one element that must to be consider: a global event like a pandemic oblige to be careful to the new ways in which phenomena we are accustomed to can manifest. Inequalities and racial matter can be exacerbated even in a multicultural context like Palermo one if social balance is missing. New form of poverty and the higher number of people exposed to this risk can give to mafia-type organizations more instrument to establish their control on the territory. The same introduction of economic measures aiming to support people can be manipulated and have a distorted effect when concretely applied. It is a great challenge because the lack of a complete comprehension can determine a failure of the adopted measures and a lack of cohesion of the social tissue. Starting from the context definition, the analysis will explore the perspective and the experiences of associations working with people belonging to the weaker part of the community, thanks to semi-structured interviews to representatives of the main local associations as privileged witness. In this way, the analysis will try to highlight how local actors' activities and how dynamics of solidarity are influenced by the global Covid-19 phenomenon.

## Introduction

Italy is the first European Country in which the Covid-19 emergency has been recorded.

The first outbreaks of Covid-19 is reported in February, in the Northern region of Lombardy (Presidency of the Council of Ministers, 2020a,b). Just few days after it, the virus reaches the Sicilian city of Palermo with the contagious of a group of tourists coming from Milan (Patané and Spica, 2020). Behind this specific case, at the beginning, Southern Italy seems to be not affected by the pandemic. Indeed, virus spreads above all in Lombardy, Veneto, Emilia-Romagna, and Piedmont. Considering its diffusion, a series of special measures are immediately adopted by government, both national and regional, in order to contain it. These measures concern the organization of health services, the definition of protocols that must be adopted if a Covid-19 case is recorded or suspected, starting to introduce limitation to the free of movement in certain area of the Country depending on the different spread of the pandemic.

Week after week, the number of contagious increases in a sort of descent toward the Southern part of the Country. Thus, measures originally applied in some Northern areas starts to be extended to the whole Country on 9th March while, just 2 days after, the 11th March marks the beginning of the so called “Lockdown,” that since that moment become a common word.

The global Covid-19 crisis shows its impact on all the aspect of life: health, social, economic, and politic. The attention mainly reserved to the economic and politic aspect musts also take into account the risk of social fragmentation. In which way this emergency has impact on communities where social fragilities are already existing? In which way, already existing social dynamics are changed by the fear of pandemic and isolation imposed by governments to contain it. In which way local associations and citizen are coping the new challenges imposed by this emergency?

The aim of this article is to reply to these questions, trying to define what Covid-19 emergency could be for our societies. Different factors are take into account such as social damage and the attempt to reorganize social relations (Corposanto, 2008, 2020; Moretti, 2020), the risk of social fragmentation and the ambivalent consequences of the increasing role of technologies in front of this new emergency (Auriemma and Iannaccone,  2020; Finch and Hernández Finch, 2020; Pagano, 2020; Zuddas, 2020). Anyway, research plan had to face on the concrete capability to reach and investigate reality. How explained in the following paragraph, interviewing representatives of civic associations working on the field have been considered as the best way to catch this particular perspective; despite of any prevision, reaching them proved harder than expected. Indeed, this represent the main limit of the work itself that could be improved extending the local actors involved.

## Materials and Methods

Considering the different spread of this emergency in different Countries, the first part of the research is dedicated to the reconstruction of what happens in Italy and in which way National and Local Governmental Institution faces on this phenomenon. In sections Pandemic Context, Criminal Dimension and Control Activities, and Economic Dimension, I propose a reconstruction of the context, considering economic and criminal dimensions, based on official data and documents released by Institutions - both at national and local level - other than articles published in the Newspapers. It aims to reconstruct the conditions in which the communities we will talk about really lives, also considering that the pandemic had a different spread in Italy and Sicily was one of the regions less involved during the first wave. Thus, data concerning the Health service conditions, the number of committed crimes, the economic performance, the level of poverty and the measures adopted by National and Regional government in this period will be considered.

In section Social Dimension, the focus shifts to the local community of Palermo, trying to explore the way in which it faces on it, through the eye of the associations working on the field.

The experiences of associations working with the weaker part of the community, in the specific Palermo context, is used to explore the social dimension and the solidarity dynamics within the community. It aims to explore a non-institutional perspective; thus, the chosen instrument are semi-structured interviews to privileged witness such as representatives of the main local associations. The list of question proposed aims to reveal how their work and activities were “before,” “during,” and “after” the lockdown imposed in Italy from 11 March to 3 May 2020. The purpose of the posed questions is to define the context before the spread of this emergency; find out in which way their activities and their user communities have been affected by the stop imposed by the introduction of the Lockdown; finally, if and in which way the association have started again the daily activities after the end of the Lockdown and what kind of perspective they have for the future.

This part would be the core of the research aiming to the discover of unofficial points of view. However, in this case the methods used are at the same time part of the result of the research itself. Difficulties found in trying to reach representatives of local associations to conduct the interviews influenced the concrete prosecution of the work and the methods that have been chosen.

In the city of Palermo, there are many associations working on the field with a specific target. The selection of the ones included in the non-probabilistic sample has been determined by the will to represent the different kind of civic associations existing. The sample included associations sharing an historical presence in the city of Palermo: Libera Palermo, Nausicaa-Andrea Ballarò, Emmaus, PerEsempio Onlus, and AddioPizzo. All of them are active in working class quarters of the city - with a target made up of people of different ages and marginalized due to economic, social, or ethnical reasons - and often cooperate together^1^. At the same time, each of them has a specific aspect: some of them are openly non-partisan (e.g., AddioPizzo) while others have a more evident political orientation (e.g., Laboratorio Andrea Ballarò); some of them are linked to national or international organization (Libera Palermo or Emmaus) while others have just a local dimension (e.g., Per Esempio). Obviously, results cannot be generalized but they can be motive for further researches and reflections.

In a different context, the research would have previewed an on-site visit at the Associations headquarters but - considering the particular context defined by the Covid-19 emergency - an email contact has been considered more appropriate. In this first written contact, motives and themes of the research have been exposed to the association offering an appointment; always considering the application of anti-Covid-19 measures, I proposed to conduct a phone interviewing too, giving the possibility to receive in advance the list of questions via mail. The results of these contacts have been the following: one of the association never replied; two of them confirmed their availability - asking for the list of question and a phone interview - but never replied to fix the appointment (neither after other prompts); the representatives of the fourth association postponed twice our fixed appointments, and the second time he did not answer to the phone call and never call back. Finally, the last representative postponed the phone interview three times; differently from the others, he always proposed a new appointment and - thanks to the researcher's determination in concluding it - it has been possible conduct at least this interview. Thus, in only one of five cases it has been possible conducting the interview.

In the light of previous experiences in social research, the problem in reaching associations is not merely determined by the Covid-19 emergency. Indeed - even if the pandemic and the necessity of maintaining social distance has as impact the reduction of the effectiveness of researcher' attempts in establishing a contact - it can be considered as an endemic problem in conducting social researches. Anyway, in a No-Covid context, I would have reach personally the headquarter of the Association and a personal direct contact would have help in establishing a direct relation with their members making easier their involvement in the research. The impossibility to reach physically their places represents an obstacle in conducting the research itself and - it can be assumed - also for people seeking their help.

Due to the difficulties in interviewing representatives of associations, more space has been dedicated to a secondary level analysis. In particular, I analyzed the contents published on the official web sites or Facebook pages of these Associations - in order to find out how they describe themselves and what kind of projects they were involved into in this period.

The presented results are discussed in the last chapter in which I proposed some items that should take into account considering the impact of Covid-19 emergency in local communities.

## Results

### Pandemic Context

After the international state of emergency declared by the World Health Organization, all flights from/to China have been canceled by government decision on 30 January. At the beginning, virus seemed far from Europe and checking of international flight was considered as a sufficient measure. The first case of Covid-19 concerning an Italian citizen - not returning from China - has been officially recorded on 21st February in the Lombardy region. Before him, a couple of Chinese tourists have been recovered with Covid-19 symptoms in the Lazzaro Spallanzani Hospital, in Rome. The pandemic affected first of all the Northern region of Lombardy and spread rapidly in the whole Northern Italy. The Country seemed divided into two parts, the North with a great number of outbreaks - determining the institution of “red zones” - and the South where the virus seemed not yet arrived.

After an increase in the spread of the emergency and in the difficulties for the Health Services to face on it, to avoid that the pandemic could affect also those areas that seemed to be more resistant, a general Lockdown has been imposed by Italian government with a Decree of the President of Council of Ministers (Presidency of the Council of Ministers, 2020a,b). In the whole Country, from 11 March to 3 April, commercial and industrial activities - not offering essential services - were forced to remain close, the use of mask was mandatory in public places and people was not authorized to get out from their homes without a proved motive (such as work, buying medicaments or alimentary stuff). In Sicily, the maximum level of contagious has been reached at the end of March and the diffusion of the pandemic here has been lower than in most of others Italian regions: 0.7 cases per thousand of inhabitants (rather than 3.9 of the national average) (Bank of Italy, 2020) and a death rate of one tenth of the national level. Despite of it, to avoid gathering of people due to national festivity, the Lockdown has been renovated in the whole Country until 3rd May.

The increasing anxiety for economic damages determined by the Covid-19 emergency pulled different group of population to evoke financial supports. The main worry concerned the summer season in which Italy, in general, and Sicily, in particular, are among favorite tourist destinations and that represents one of the most important economy sector. Due to the need to start again and an effective reduction of cases, since the end of Lockdown period the emergency seemed to be dramatically reduced. Wearing the mask and adopting basic hygienic measures were still necessary but the reopen of commercial activities, restaurants, museums and similar activities gave people the idea that the worst period was behind. Weddings and religious events were possible again and people restarted to move from a region to another for the summer vacation.

In these days, we are aware that it was just an illusion and that, probably, the defenses were lowed too early.

The following paragraphs will explore the different dimensions on which Covid-19 emergency - and the consequently measures adopted in contrasting it - have had impact.

### Criminal Dimension and Control Activities

The respect of anti-Covid-19 measures implied a dramatic restriction of the free movement of people and an increase in the presence of Police force and Army too in order to control the respect of those measures. According to the *Dossier Viminale 1 agosto 2019 - 31 luglio 2020*, in the period 11 March - 3 May, 12,360,197 people have been controlled and among them: 418,222 have been denounced for having violated the limitation of free movement, 5,280 for having given false information, and 886 for having violated the quarantine period (Dipartimento della pubblica sicurezza Minister of Interior, 2020a). Fire department has been greatly involved in the Covid-19 emergency in sanitization activities of public places, support activities to the transport of urgent instruments, to civil protection, to hospitals and local administration in giving information (Dipartimento della pubblica sicurezza Minister of Interior, 2020a).

The dramatic change in daily life had an impact on the criminal dimension too, in terms of reduction of numbers of committed crimes. Analyzing the period between 1 and 22 March, the Italian Department of Public Security (Dipartimento della pubblica sicurezza Minister of Interior, 2020b) finds out a reduction of the 64.2% of committed crimes in the whole national territory (146,762 in 2019, 52,596 in 2020). According to this report, the reduction is not completely homogeneous considering the different area of the Country and the specific type of crime. A strong reduction has been recorded as far as concerns the exploitation of prostitution (−77%), burglaries of postal offices (−73.7%), home burglaries (−72.5%), and sexual abuses (−69.9%). On the other hand, other kind of crimes register less reduction such as domestic violence (−43.6%) and burglaries of pharmacies (−24.6%). Clearly, these are operational data and cannot include the whole number of crime committed; considering the domestic violence, for instance, difficulties for the victim in concrete denouncing suffered abuses being obliged to remain home with the persecutor himself must be take into account.

Concerning the different areas of the Country, the regions in which a higher reduction of crimes has been recorded are Lombardy and Veneto that are also those where anti-Covid measures have been applied firstly because of the higher number of cases. Despite of this general statistic reduction, Sicily is one of the areas^2^ in which the number of crimes that arouse the greatest social condemnation (robberies, damage, wounding with intent, computer fraud and burglaries) remains higher than in other regions. The city of Palermo has to face on economic, social and criminal problems. Its history is characterized - and has been determined - by the presence of the mafia-type organizations since the XIX century. This phenomenon affects not only the economic dimension but the social too and this is the reason why it is so difficult to fight. Anyway, the fight against it has a long history too and the merit must be recognized to those Authorities, Institutions, Associations and common citizens who are daily engaged. Indeed, the pandemic did not stop the activities of Police and Magistrates. In the last few months several were the police operations, result of long investigations, leading to the arrest of teens of member of mafia groups such as the “Teneo” and the “Stele” operations, conducted by the Carabinieri of Palermo in June and in July 2020 (Giornale di Sicilia, 2020b). Moreover, the “Mani in pasta” operation conducted in May 2020, has been really realized in the midst of Covid-19 emergency. This operation lead to the arrest of 91 members of mafia groups and the sequester of their assessment (valued for 15 million of euros) involving not only Sicily but Lombardy, Piedmont, Liguria, Veneto, Emilia-Romagna, Tuscany, Marche, and Campania too (Il Fatto Quotidiano, 2020a). The investigation of Police and Magistrate confirms the potential role of Mafia during the Lockdown in offering (a dummy) help to face on the crisis determined by the closure of commercial and industrial activities. Indeed, for a lot of people the measures adopted for the containment of the emergency had as consequence the loss of job, money and the ability to feed themselves behind a liquidity crisis for the entrepreneurs. As stated by the Judge Morosini, these conditions give space for usury, money-laundering and racket (Il Fatto Quotidiano, 2020a) and just in these days, in Catania, Finance Police arrested in flagrante delicto a young usurer (linked to mafia groups) for a loan with an interest rate of 120% to the owner of a restaurant in crisis for the Covid-19 emergency (Il Fatto Quotidiano, 2020b).

### Economic Dimension

The forced closure of a great number of economic activities - introduced with the Lockdown - determines a vicious circle that reduces entrepreneurs' revenues leading them to difficulties in paying the rent of offices and shops; owners of these offices, in turn, will have economic difficulties due to the nonpayment, and so on. When the pandemic started nobody was able to define the real damages that it would have been determined - probably neither then, nor now.

In the Cerved Industry Forecast released on March 2020 (Cerved Industry Forecast, 2020), one of the main Italian actor in the analysis and credit risk management proposed two scenarios. The first one assumed the hypothetical end of the emergency in May 2020 with other 2 months for a complete return to normalcy. Obviously, it is not the case, thus the second scenario is the more credible: pandemic would end at the end of the year 2020, behind 6 months for a return to normalcy. According to this second hypothesis, entrepreneurs would have a loss of revenues of the 17.8% (also considering that this year an increase of 1.7% was previewed); in 2021, there would be a great increase of 17.5% that would be not sufficient to regain the 2019 level. The economic sectors most affected by the crisis would be the hotel industry, the travel agency and the extra hotel accommodation one for which the study estimated a loss of −73.3, −68.8, and −64.2%, respectively. In Sicily, touristic sector (including accommodation and food services) is the one showing a higher vulnerability because it highly depends on the foreign demand based on travelers' confidence that takes longer to be restored (Bank of Italy, 2020). Due to the closures imposed, occupancy rate recorded a reduction too between March and April even if it has been mitigated by the great access to the Wages Guarantee Fund and the block of layoffs disposed by national government. Local Institutions themselves have to face on economic difficulties (already existing before the spread of Covid-19): due to the lockdown, tax revenues for Sicilian municipalities are expected to record a reduction of the 48% of annual revenues (the national average in this case is 60%) (Bank of Italy, 2020).

In 2021, in no region in Italy the turnover of contractors will be able to regain pre-Covid level. Indeed, in 2020 Sicily would register a reduction of 18.3% than 2019 while the increase estimated in 2021 comparing to 2020 is of 17.9%; considering these variations, the revenue estimated in 2021 is lower of 3.6% than 2019.

Anyway, the context is not uniform and homogeneous. Indeed, the pandemic caused the closure of some kind of activities with loss of revenues; at the same time, other kind of activities recorded an increase of work such as supermarkets, health services, delivery services, cleaning companies specialized in sanitization. Still according to the Cerved study, some activity sectors will reach great percentage of increasing: e-commerce 55.0%, modern food retail 22.9%, wholesale of pharmaceutical and medical goods 13.8% (Bank of Italy, 2020). Moreover, behind politic critical and instrumental debates, the National Government has introduced a series of economic measures in order to support the local economy, both the user and the entrepreneurs, such as: a one-off contribution to the owner of commercial activities, the so called “Bonus Vacanza” to help tourism recover, the government intervention on the redundancy fund, behind those measures applied by regional government.

### Social Dimension

During the Lockdown, new form of solidarity emerged. The extraordinary measures adopted by government and the data concerning the critical conditions of the Health Service, determined the increasing of donations from citizens, organized and promoted by non-profit organizations, hospitals, and influencers (Italia non profit, 2020). In front of the increasing number of fundraising, it must be noticed the initiative promoted by *Italia non profit* and *Associazione Italiana Fundraiser* (Italian Fundraiser Association). Aiming to guarantee transparency and data access both to donors and beneficiaries, the two associations created a dedicated web page on which all the official fundraising actually active to sustain specific hospitals - in the whole Country - can be found^3^. Another interesting project is the *Solidarietà Digitale*, an initiative promoted by the Italian Minister for Technologic Innovation and Digitalization^4^. In the light of the imposed Lockdown, it aims offering free services to people, professionals and companies supporting them in smart-working, providing access to newspapers and books by smartphone or tablet; providing access to e-learning platforms, medical consultants, or allowing distance activities such as sporting activity, hobbies, etc. Looking at informal initiatives, a thought must be dedicated to the flash mobs that every day, twice per day, saw people leaning out from their own house's balcony or window to play music or strike up a song; it became a way to not feel alone even if obliged to stay home. Behind these forms of solidarity, the health emergency manifests the capability to exacerbate inequalities and discriminatory dynamics. Recalling data released by the U.S. Bureau of Labor Statistics in 2018, a study conducted on the relation between poverty and Covid-19 in the U.S. (Finch and Hernández Finch, 2020), the risk of marginalization is higher for those people with lower level of education and a lower capability to work from home; in a vicious circle, those who have more difficult in working from home due to the technological gap are forced to work outside exposing themselves to a higher risk of infection.

Comparing to other area (both in Italy and abroad), Sicily has a complex reality concerning socio-economic and criminal dimensions. Mafia phenomenon has spread its presence all over the world, since a long time and in different forms too, but Sicily is surely one of the region more affected by it and its consequences. Criminal and economic conditions of this region have an impact on social dimension already in normal situation: in the region, the level of income is lower than the national average while there is a higher inequality in its distribution. In the multi-dimensional crisis determined by the Covid-19 emergency, common difficulties in daily life are amplified and there is a concrete risk of exacerbating social inequalities and increasing the quote of families living in conditions of absolute poverty, already higher than the national average (Bank of Italy, 2020). Moreover, according to the last Istat Citizens, Businesses and ICT report (2018) the percentage of Italian families having access on internet from their own home is lower than European ones and the gap in terms of digital infrastructure divide is highly manifest in the lines of inequalities already dividing North- South or city-countryside (Auriemma and Iannaccone,  2020). In this paragraph, the scope is to bring out the concrete experience and point of view of civic associations working in the field that must daily face on the interlink among social, criminal, and economic dimensions.

As described in the section “Materials and Methods,” contacting local associations has been more difficult than previously assumed. In this period, most of offices and services can be reached just by appointment to avoid gatherings of people. As result, it has been preferred a previous contact via mail rather than joining personally the associations' headquarters. In this sense, the first result of the study is the lower capability to reach associations in a context in which social contacts are necessary reduced.

As exposed, the five contacted associations have been chosen considering the kind of activities they realize, their users target, and the structure itself in order to provide a better perspective and a wider horizon. Among the contacted associations, just the representative of AddioPizzo was finally disposable to conduct the interview, even if also in this case it has been necessary scheduling a series of appointments due to his numerous work commitments. The problems encountered determined the choice to consider as second instrument of analysis the communicational channels used by associations themselves to promote their activities; in this way, the research aimed to offer a panorama as fully as is possible. Thus, the following paragraphs present the different element of analysis considered, distinguishing among the situation perceived *before, during*, and *after* the lockdown determined by the Covid-19 emergency in the city of Palermo^5^.

#### Before

Here a brief description of associations and their main activities before the spread of Covid-19 emergency.

*Per Esempio Onlus*^6^ is a non-profit association created in Palermo, in 2011. Their activities take place in some of the most popular quarter of the city such as Ballarò, Borgo Vecchio, and Zen and they are focused on: contrasting educational poverty, school dropout, and social inclusion; promoting volunteering and active citizenship; promoting integration of migrants and meeting between cultures; promoting European mobility in a life-long learning perspective.

The *Laboratorio Andrea Ballarò*^7^ is a Cultural Center - adhering to the network of Associazione Ricreativa Culturale Italiana^8^ (ARCI) inspired to democratic values emerged in Italy to fight against nazi-fascism. The Association organizes Cinema-Forum, and public debates on socio-political issues.

*Emmaus Palermo* is an association adhering to the Emmaus Movement and is inspired by its ideals and values. The association's scope is the promoting of volunteering activities in favor of all groups of population - both Italian and not - who experience suffering for social, economic, and cultural disadvantage, including people of all the age, gender, political orientation and nationality. The Emmaus community in which these people are welcomed aims to self-sufficiency thanks to the sale of second-hand products result of people donations, organizing activities promoting human solidarities, and organizing summer camps for volunteers in reused confiscated assessment.

Libera Palermo and AddioPizzo are probably the most famous among the contacted associations. *Libera Palermo*^9^ is one of the Local Coordination Committee of the National *Libera. Associazioni, nomi e numeri contro le mafie*, and it is officially active since 2008. The aim of this Local Committee is to apply in the specific context of the city of Palermo the values of the National association. Thus, the main issue is the promotion of a critical consciousness in front of mafia phenomenon and its evolution, in combination with the engagement in contrasting educational poverty, supporting young people, supporting mafia victims' families, promoting the social reuse of mafia confiscated assessments.

*AddioPizzo*^10^ was born as Movement in 2004 by a group of young who posted all over the city of Palermo the now-famous statement “Un'intero popolo che paga il pizzo è un popolo senza dignità” (“An entire people that pays protection money is a people with no dignity”). After it, the same group created the homonym association of volunteers, without political orientation, with a specific connotation of being promoter of a virtuous economy aiming to be mafia free. The channel chosen as instrument is the promotion of critical consumption - referring to producers and traders adhering to the association itself - and the support to victim of racket. Even if it is now largely famous and proposes their outreach activities in the whole Country - and abroad too thanks to the AddioPizzo Travel agency - AddioPizzo is deeply rooted in its territory. In the last years, the association have implemented projects on social inclusion and urban regeneration projects in the popular quarter of *Kalsa* in which the association has its headquarter. Projects on educational and economical poverty are realized in cooperation with other local actors such as the *Massimo Theater*, the Botanic garden, the CONI (the Italian National Olympic Committee) and the educational institute of the quarter.

#### During

On 11 March, like other activities, all the associations considered have been forced to cancel or reorganize activities and projects overnight. According to AddioPizzo representative's words, the main consequence of the suspension of activities and the oblige to stay home is the suspension of the physical presence of volunteers in the quarter. After an initial disorientation, the association has been successful in maintaining contact with users and their families. Considering the technological gap of this group of population, they requested and obtained the authorization from local Authorities to give technical support to people in the request of food subsidies specifically introduced for the Covid-19 emergency. Considering the bureaucratic procedures and aiming offer a quicker service, the group decided to organize other types of services integrating the different group of users whom their activities are usually addressed to. Thus, they started to collect food and ready meals - produced by traders adhering to the AddioPizzo network of critical consumption - and distribute them to the quarter population. People were advised by phone and this allowed the association to get in touch with almost 150 families, continuing their social inclusion activity. This show one of the most important element emerging from the analysis of reality that is the necessity of a redefinition of scopes, instruments and strategies in order to cope the new challenge determined by this emergency.

Trying to explore the people reaction to this new condition, AddioPizzo representative talks about desolation, discouragement, anger, and concern as emerging feelings within community. At the same time, an increasing appreciation seemed to emerge in front of the activities conducted by volunteers. Also in the representative's words there is a clear reference about a perceived reduction of crimes corresponding to the official data released by Authorities. Anyway, the scenario described is a context with economic, social, and urban complexities exacerbated by the pandemic: “people is no hungrier than before but we see more evidently a deal of discomfort and…people who more than ever live from day to day, and who are not able to look beyond due to this context….” The activities made up by the association are appreciated but seem not being sufficient in front of people needs and worries.

Maintaining contacts with people in the respect of social distancing implies the use of different kind of instruments and reveal the power of technology in connecting people. Instruments like YouTube Channels, Skype, Facebook, and Teams allowed associations to pursue their activities - both internal and with users (think of the listening activities in supporting victim of extortion) - and make them known. At the same time, these instruments are less useful for organizing activities with those groups of population among which the digital illiteracy is highly diffused. The technological gap existing within the community reduces the utility of this instrument to maintain contact between associations and users during the lockdown considering that most of them have not basic technological knowledge nor technological devices.

#### After and Future Perspectives

Starting from 3 May, the gradual reopening has been disposed by National Government determining the end of the Lockdown period.

AddioPizzo association has not been able to restart all its activities on the field, above all those strictly linked to the opening of schools. Volunteers are present in the field again but not all kind of activities can be restarted. Here again it is possible distinguishing among the different type of activities of the association and their target. If laboratorial activities outdoor or fun activities close at hand could have been organized in the main square of the Kalsa quarter, it was not possible organize sport activities implying a physical contact among player considering the impossibility to maintain the respect of anti-Covid measures. Moreover, fear of families in letting their children participate in social activities must be taken into account: “Families continue to be worried and, in some cases, they decided to maintain home their children.” There is a great desire to restart with normal activities, above all among young people, putting all this behind even if - the representative admits - “the time is not ripe.” Considering the activities in supporting victim of extortion, it has been already underlined that Covid-19 has not stopped them. The Association restarted to accompany denouncer to police authorities; a concrete proof is the last police operation conducted in *Borgo Vecchio*- one of the most popular quarter of Palermo - that have seen a collective denounce of local entrepreneurs (Giornale di Sicilia, 2020a).

In other words, there is a great will to start again but - like any other local actor - the Association and their future perspectives are conditioned by the effective prosecution of pandemic.

In a positive scenario, the association is willing to organize projects able to integrate the two types of activities already pursuit: social involvement of young people and support to local entrepreneurs in facing extortion and organized crime's influence. In this sense, they will aim to create a positive contamination thanks to the involvement of businessman adhering to the AddioPizzo association in training programs for young. Actual situation does not allow to consider the immediate future in a positive way. Actually, the pandemic is not stopped and European Countries are experiencing the second wave. In Italy, restrictive measures have been reintroduced both by national and local governments and starting from 24 October it is clear that the situation is not solved: even if it is not still mandatory, people are highly recommended to stay home; Health Service is day after day more stressed and other more restrictive measures are expected in the following weeks.

## Discussion

This research aimed to explore solidarity dynamics in the new context determined by the Covid-19 emergency in the Palermo context. The context has been defined according to criminal, economic, and social dimensions giving room to the direct experience of civic associations. Analyzing the impact of Covid-19 emergency on community's life in Italy, it is nowadays possible distinguishing two phases, corresponding to the two waves of the emergency itself.

In the first one, everyone was astonished by the unexpected panorama defined by the spread of virus. Behind conspiracy theories, nobody was prepared to face on a situation of this type and - in front of a common and unknown enemy - a sentiment of union invaded everything. Different forms of solidarity emerged, from the increase in donation to the play of music from balcony at 6 p.m. to feel less alone. Some more than others, everybody accepted the application of limitation to free of movement and the closures applied to activity sector. In this phase, the pandemic seemed to reinforce unexpected social links.

Summer represented a transitional moment. The emergency seemed to be completed, the first financial aids were arrived, commercial and social activities started again, and restrictive measures were gradually relaxed. Someone talked about a possible second wave but the worst seemed to be behind, above all in Southern Italy.

The second phase started in October, with increasing numbers of cases recorded in the whole Europe. In the Italian case, two new elements can be retraced. First, Sicily - and Southern Italy in general - is now affected by pandemic like other Italian regions; second, a sentiment of disagreement is now evident in the whole Country. In many Italian cities, groups of people manifest against the new measures applied (above all the closure at 18:00 of bars, pubs, and restaurants) and most of these events are infiltrated by violent groups; it happens from North to South in important cities such as Milan, Turin, Trieste, Naples (RaiNews, 2020a), and in Palermo too (RaiNews, 2020b). In this phase, solidarity dynamics seems to have taken another route and the initial sense of unity left room to individual claim. Subjective dimension has here an important role considering that the way in which the pandemic emergency is interpreted varies according to social variables such as the age, the civil status or the specific moment of life that the person himself is experiencing. In other words, solidarities dynamics that can be retraced at the beginning of the emergency risk to be clouded by the personal claim of freedom and the specific conditions experienced by different groups of population.

In general, the Covid-19 emergency showed all the Institutional weaknesses of a Country like Italy where technological gap, poverty, gender discrimination, and different level of economic development are already present. Simply, in an emergency like the one we are living the chickens have come home to roost. In this sense, Palermo is a community where social fragilities were already existing and in which the Covid-19's impact can be retraced in their exacerbation if local actors are not able to redefine their strategies and way of doing like AddioPizzo Association did.

In the light of the above, three main issues should be take into account.

1. *Social Fragmentation and Inequalities*. During the pandemic technologies showed their powerful role helping people maintaining social relations, enterprises and associations continue their activities thanks to the smart working, scholars continue their lessons, public system itself had to offer services via web in order to avoid gatherings, and people can keep in touch with their hospitalized parents (Moretti, 2020). At the same time, the necessity to use different instruments makes the technological gap among different population groups more evident. Considering both the digital infrastructure divide and the cultural one (Auriemma and Iannaccone,  2020), the existing inequalities have been exacerbated by the Covid-19 emergency determining the risk of social discriminatory, inequalities and marginalization effects in local communities (Pagano, 2020). It is evident in working sector but in education too: in order to work or study from home, each member of the family should have a dedicated space, an its own device and a very good web connection; moreover, children should be accompanied by an adult that possibly should work at the same time (Pagano, 2020). Indeed, the health emergency determines a democratic matter considering that a large part of society is not able to use a device - nor applications or web services - or have access on Internet. Moreover, the interview to the representative of AddioPizzo Association shows also that technologies cannot substitute entirely direct contact with people where physical presence is the main element of the interaction itself.
  The events of the last weeks confirm how fragile is the equilibrium within societies. In a long-term perspective, Covid-19 emergency divides people. Not only because of the physical isolation or the cited technological gap but for the individual reaction concerning the measures applied by government. The main element that provokes animosity is the limitation of personal freedom and the higher level of social control. This division risks to be manipulated and used by different actors - both political actors and groups of extremists - to gain more power or simply to destabilize the situation. The debate itself - on the way in which the management of emergency is considered - is able to provoke a rupture within the community.
  It is necessary to recover the sense of unity experienced during the first lockdown period to avoid the concrete risk of social fragmentation and inequalities. The physical distance must be maintain promoting social proximity (Corposanto, 2020).
2. *Quality of Life*. In a place where a mere handshake is part of social relations, the application of the minimum level of anti-Covid-19 measures aiming to avoid a direct contact among people - such as wearing a mask and maintain physical distance - can have a great impact on social life (Corposanto, 2020). During the lockdown, the impossibility to get out from their own homes implied the impossibility for members of the same family to meet each other and the lack of physical contact is something to which people were not used to. Behind a possible lockdown, the high number of asymptomatic people and the high contagious power of the virus force authorities to advise people staying home not having contact above all with vulnerable subjects of the family itself. The social damage determined by the deny of social relations due to the illness is what Cleto Corposanto defines as *Sonetness* that leads to the necessity of finding an equilibrium between social damages and economic ones (Corposanto, 2020). If economic damages caused by the pandemic are evident, the contraction of number of crimes is something that must be notice too. This positive element must always be considered like a result of the corresponding decrease in freedom of citizens. Less people can freely walk in the street, less people can be victim of bag-snatching; obviously, not this is the way to improve quality of people' life. The reduction in committed crimes should be the result of successful policies in contrasting and preventing them and not the result of decreasing citizens' rights and freedom.
  At the same time, members of communities should define what does quality of life really mean and what are the priorities to follow. Being required to wear a mask in public places is really an attack on our Constitutional rights? Tracing our contacts if we are Covid-positive is really an intrusion in our personal life when every day we give our data to multinational companies just surfing the internet? Most of the issues community must face on now are the same big issues of the last decades. Indeed, issues such as the weaknesses of the Healthcare system, the lack of digital skills among small enterprises, or the weaknesses of working sector are the results of mismanagement in which corruption and personal interests have had an important role. We should stand up yesterday more than today.
3. *Redefinition*. The last issue that emerges from the analysis proposed is the necessity to redefine scope, instruments, and strategies. If reality changes our way of doing must change too, re-formulating activities in order to answer to new needs. The first solution has been identified in reducing the digital divide. At the same time, the Covid-19 emergency forces us to face the facts being aware that solutions cannot be so immediate. For instance, the technological gap evident in determined groups of population cannot be solved overnight but it requires investments both in infrastructure and in digital alphabetization. At the same time, in the working field, a massive application of smart working could have relevant contraindications such as more costs charged on workers, limited occasions to share experiences and knowledges that could obstacle workers' capacity to organize and claim for better working conditions, or the overlapping between private and working life (Zuddas, 2020). Considering technologies as unique solution means focusing the attention on the risk of digital marginalization forgetting the “pure” social one already existing (Zuddas, 2020). Thus, it is possible to find other kind of instruments or ways to avoid the risk to be marginalized? It is an extraordinary situation for modern societies and the challenge is great. The pandemic forces governments to apply measures limiting freedom of citizens, both in economic field and in social life. At the same time the border between social security and the Constitutional respect of citizens' rights is unstable. Western democratic system is put to the test but the Covid-19 emergency could be the trigger to improve our life.

In other words, in front of the actual risk of new *social fragmentation and inequalities* determined by the Covid-19 emergency, communities should re-conceptualize what *quality of life* really is and *redefine* instruments and strategies in order to reach it.

## Data Availability Statement

The original contributions presented in the study are included in the article/supplementary material, further inquiries can be directed to the corresponding author/s.

## Author Contributions

The author confirms being the sole contributor of this work and has approved it for publication.

## Conflict of Interest

The author declares that the research was conducted in the absence of any commercial or financial relationships that could be construed as a potential conflict of interest.

## Footnotes

^1^See the letter to the President of the Italian Council concerning the collective denounce lodged by local businessmen in Palermo, against extortion episodes: https://m.facebook.com/moltivolti/photos/a.227967570733907/1518845184979466/?type=3&source=57. See the claim to the President of the Region of Sicily denouncing the risk of mafia expansion due to the pandemic: https://palermo.repubblica.it/cronaca/2020/05/06/news/coronavirus_la_crisi_puo_favorire_la_mafia_venti_aziende_e_associazioni_scrivono_all_antimafia-255831282/?rss.

^2^The higher number of crimes committed in the period considered are: Lombardy (8.541), Campania (6.208), Lazio (6.164), Sicily (4.665), and Piedmont (4.354).

^3^For further info visit: https://italianonprofit.it/donazioni-coronavirus/.

^4^For further info visit: https://innovazione.gov.it/coronavirus-solidarieta-digitale-in-tutta-italia/.

^5^In the following pages, statements within quotation marks refer to AddioPizzo representative and have been personally translated.

^6^See more at: https://peresempionlus.org/.

^7^https://www.facebook.com/Laboratorio-Andrea-Ballar%C3%B2-108159523963605

^8^https://www.arci.it/

^9^http://www.bottegaliberapalermo.it/

^10^https://www.addiopizzo.org/
